# Supplementary material for: Patterns of Intron Gain and Loss in Fungi
Source: PLoS Biol. 2004 Nov 30;2(12):e422. doi: 10.1371/journal.pbio.0020422 (PMC532390; doi:10.1371/journal.pbio.0020422)
Supplement: Table S1 — Also available at http://genes.mit.edu/NielsenEtAl/. (4.3 MB ZIP). [file pbio.0020422.st001.zip › NielsenEtAl/html/862.html]

AN3176.1.NCU03380.1.MG02417.1.FG10896.1


```
 CLUSTAL W (1.82) Multiple Sequence Alignments - Introns Inserted


Sequence 1: AN3176.1	638 aa
Sequence 2: FG10896.1	637 aa
Sequence 3: NCU03380.1	654 aa
Sequence 4: MG02417.1	636 aa
Alignment Length: 660 aa
Number Identitical Residues: 255 aa
Alignment Score (without introns) 13696


MG02417.1 	MAPEKAPQKFS---RSWDALTPPLADFILEAVASMGFNELTPVQAATLPLFRGNKDVVVE
NCU03380.1	MAPTQPQTKVRKPPRSWDALTPPLAQWILDYLSSMGFTQPTPVQKSCLELFRGNKDVVVE
FG10896.1 	MATEKPKKRSP---RAWDTLNPPLSEWIRDAVATMGFDQMTPVQAATLPHFMGNKDVVVE
AN3176.1  	MAPKPPSGTSS---RAWDAVNPPLSEWVLDAVSSMGFTRMTPVQASAIPLFMAHKDVVVE
          	**.  .        *:**::.***:::: : :::*** . **** : :  * .:******

MG02417.1 	0AVTGSGKTLAYLI1PIID~KILRRDDVLKRHHVGAIIVSPTR~ELATQIHSVLSSLISF
NCU03380.1	~AVTGSGKTLAFLI~PVVE0KLLRGEEPAKRNHVQGIIISPTR~ELATQIYNVLVSLVKF
FG10896.1 	0AVTGSGKTLAFLI~PLVQ~KLLRLSEPTKKHHVAAIIVSPTR~ELAAQIHTVLMKLLQF
AN3176.1  	~AVTGSGKTLSFLI~PVVE~KLLRLEEPIKKHHVGAIIVSPTR2ELASQIYNVLTSLLAF
          	 *********::** *::: *:** .:  *::** .**:**** ***:**:.** .*: *

MG02417.1 	HAPSAEFSSFLKG-DEKRP--DTIVPVLIPQLLVGGTVKVAQDLSTFLRLSPNILVGTPG
NCU03380.1	HEPSAEAISHAKS-DEKRP--TATQPVVVPQLLVGGTTKAAEDLGTFLRLSPNLLIGTPG
FG10896.1 	HEASAEILPHLKDDDEKRP--FTTVPAIVPQLLVGGTTTTVQDLRFFLRHSPNVLISSPG
AN3176.1  	HPASAAVINTSETEDVPRPKHSSSVLRVVPQLLLGGSTSPAEDLSTFLKRSPNLLVATPG
          	* .**      : .*  **.  :    ::****:**:.. .:**  **: ***:*:.:**

MG02417.1 	RLSELLSSPYVHTPQSSFEVLVLDEADRLLDQGFQKELQRILGFLPKQRRTGLFSASVSD
NCU03380.1	RLAELLSSAYVKAPASTFEVLIMDEADRLLDMGFANELNRILGYLPKQRRTGLFSASLSD
FG10896.1 	RLVELMSSPHVHCPQSSFEVLVLDEADRLLDLGFKPDLQKILSHLPKQRRTGLFSASVSE
AN3176.1  	RLLELLSSPHVYCPQSSFEMLVLDEADRLLDLGFKETLQNILRRLPKQRRTGLFSASVSE
          	** **:**.:*  * *:**:*::******** **   *:.**  *************:*:

MG02417.1 	AVGELVRAGLRNPQRIAVTVKRLTDGGVIEDRKTPAS~LQMSYLSVPASQKWPALIQFLR
NCU03380.1	AVERLITVGLLYPHKITVRVKSLKDGGIIQERKTPMS~LQMSYLVTPASQKMPAIVQLLE
FG10896.1 	AVGEIIRVGLRNPVKIEVKVK-IKGGGILEDRKTPAS~LQMTYMVKPASQKLPALAELLR
AN3176.1  	AVDQIVRVGLRNPVKVVVKVK-GASG--VDDKRTPAS2LQMTYLTQPPTGKFPALKHILN
          	** .:: .**  * :: * **   .*  :::::** * ***:*:  *.: * **: .:*.

MG02417.1 	KVEPTPLRTIIFVSSCFAVK2YFHAVIASLLPKDVSLIPLHGKQEPQVREKCFDRFVGSS
NCU03380.1	KLEPRPQRSIIFFSSCMAVK~YFSRILGAVLPAGFSVTSLHGKLEPKVREKNYERFVNTT
FG10896.1 	QLPVRPQRSIVFLSTCAAVD~YFQHILPLILPEGFALVPLHGKHAAKVREKNFNKFLSSV
AN3176.1  	SVQPTPSKSIFFVSTCSGVD~YLSVILPLILGNDFQLIPLHGKHPANVRQKNFNRFVNAH
          	.:   * ::*.*.*:* .*. *:  ::  :*  .. : .****  .:**:* :::*:.: 

MG02417.1 	SPSILLTTD1VAARGLDIPQVDVVFQFDPPSDPKTFVHRAGRAGRAGRRGLAVALLLPGR
NCU03380.1	SPMVLLTTD~LAARGLDIPQVDLVIQHDPPTDTKVFIHRCGRAGRAGRRGLAVVLLQPGR
FG10896.1 	SPTILLTTD~LAARGLDIPQVDLVVQIDAPSDPKVFIHRSGRAGRAGRKGLAVVMLHPGR
AN3176.1  	NPAILLTTD~VASRGLDIPSVDLVVQIDPPSDPKTFIHRCGRAGRAGRRGLSVVLLHPGR
          	.* :***** :*:******.**:*.* *.*:*.*.*:**.********:**:*.:* ***

MG02417.1 	EQDYVGFLEVRKTPITPLTNPEIKVSDEEVKLFAAQVRKQATADREVFQLSQRAFVSWAR
NCU03380.1	EEGYVQLLEVRQTPITPLEKPAISVTPDEVERVSAAFRSQALADREIFQMAQLAFVSWTR
FG10896.1 	EEDYVQFLEIRKTPIAPLEKPTITTSEDDAAEFAKKTRDFVLTDRGLFDKAQKAFVSWAR
AN3176.1  	EEDYVSFLEVRKTPVAPFPHP-ITVSDAEAAAATETARKVVKADRAIHDRGQKAFVSWLR
          	*:.** :**:*:**::*: :* *..:  :.   :   *. . :** :.: .* ***** *

MG02417.1 	SYIEHKATSIFRITDVDWLDTAQGWGLVTLPKMPELKGSGIDRSLGHGIDIESIPFKDKA
NCU03380.1	SYIEHQASSIFRIADLDWLDLAKGYGLLELPKMPEVREAKIDRSLGLGINTEEIPFKDKV
FG10896.1 	SYGAHQATSIFRAADLDWADLGNAWGLLRMPRMPELKGWTGDKMCGLEIDWDNYAYKEKT
AN3176.1  	SYSKHQASSIFRVADLDWEGLGKAWGLLKLPKMPELKNFKGDKTLGVQMDWDTYAYKDKQ
          	**  *:*:**** :*:** . .:.:**: :*:***::    *:  *  :: :  .:*:* 

MG02417.1 	KEKKRQEELAQAEEDRKNGVLSAKAAALAAKRKNNEAWSGRQGQEDTRTERRERKQRKRE
NCU03380.1	REKKRQEELAKWKEEK---AKRAQEENTGDKRKKNEAWSGKAEQEETKLQRREKKRRKRE
FG10896.1 	REQQRKVALEEEKSGV---KKQDKSEEFKRKRKNNEAWSAKHEKEDDRVERREKRRKRRD
AN3176.1  	REKRRLELLQEMAESG---QQQTTN---KKRPNETVAWSNNAENRNKKAKRRDMKQVRQE
          	:*::*   * :  .                : ::. *** .  :.: : :**: :: :::

MG02417.1 	AVKVSKMTDEEKAEQKRLEDMIAEVRRRN------QEAAALASK-----------DGGDD
NCU03380.1	AKKFSEMTEKEKEEHLKLEQMIEEVRKRNEAKAAEERAAALAANPPKPKAPVKKVDDSDE
FG10896.1 	AEATSKMTDDEKVKQMELNDLIAEVRRQNREKAAAEAAAAKQEK--------DGE-----
AN3176.1  	RKRWEKMTEEEKKKALETEQMLEQIRAKNEEQRRLKRAAAKADK--------DAEEGGDE
          	    .:**:.** :  . :::: ::* :*  .   : ***   :        .  .....

MG02417.1 	GFEGFD-
NCU03380.1	EFGGFDD
FG10896.1 	-FKGFDD
AN3176.1  	EFTGFD-
          	 * ***
```
